# [Corrigendum] Elevated microRNA-125b levels predict a worse prognosis in HER2-positive breast cancer patients

**DOI:** 10.3892/ol.2026.15928

**Published:** 2026-10-29

**Authors:** Yanwei Luo, Xinye Wang, Weihong Niu, Heran Wang, Qiuyuan Wen, Songqing Fan, Ran Zhao, Zheng Li, Wei Xiong, Shuping Peng, Zhaoyang Zeng, Xiaoling Li, Guiyuan Li, Ming Tan, Ming Zhou

Oncol Lett 13: 867-874, 2017; DOI: 10.3892/ol.2016.5482

Following the publication of the above paper, and an Expression of concern statement (doi 10.3892/ol.2026.15765) that was published to report both that panels 2a and 2b in Fig. 2 contained an overlapping section of data in spite of the different degrees of staining reported for the panels, and that certain of the data in Fig. 1A were strikingly similar to data which subsequently appeared in a paper written by different authors at different research institutes in the journal *Molecular Medicine Reports* (and which has since been retracted from that publication), the authors have now responded to the queries raised by the reader and the Editorial Office. Regarding the second issue, the authors were able to satisfactorily demonstrate that the data presented in Fig. 1 were obtained from their own experiments. Concerning the overlapping sections of data in Fig. 2, panels 2a and 2b, the authors acknowledged that the two images exhibited a partial overlap in the captured fields of view. These images were intended solely to show qualitative examples of staining grade changes, and were not derived from different experimental treatments or independent biological replicates. Nevertheless, the authors have provided new representative images for Fig. 2Ab (showing “weak” staining) and Fig. 2Ad (showing “strong” staining), which accurately reflect the staining intensity grading criteria as they were originally intended, and the revised version of Fig. 2 is shown on the next page.

Note that the replacement of this data panel does not alter any of the statistical analyses, the data interpretation, or the scientific conclusions of the study. All the authors agree with the publication of this corrigendum, and the authors are grateful to the Editor of *Oncology Letters* for allowing them the opportunity to publish this. The authors apologize to the readership for any confusion caused.

## Figures and Tables

**Figure 2.**
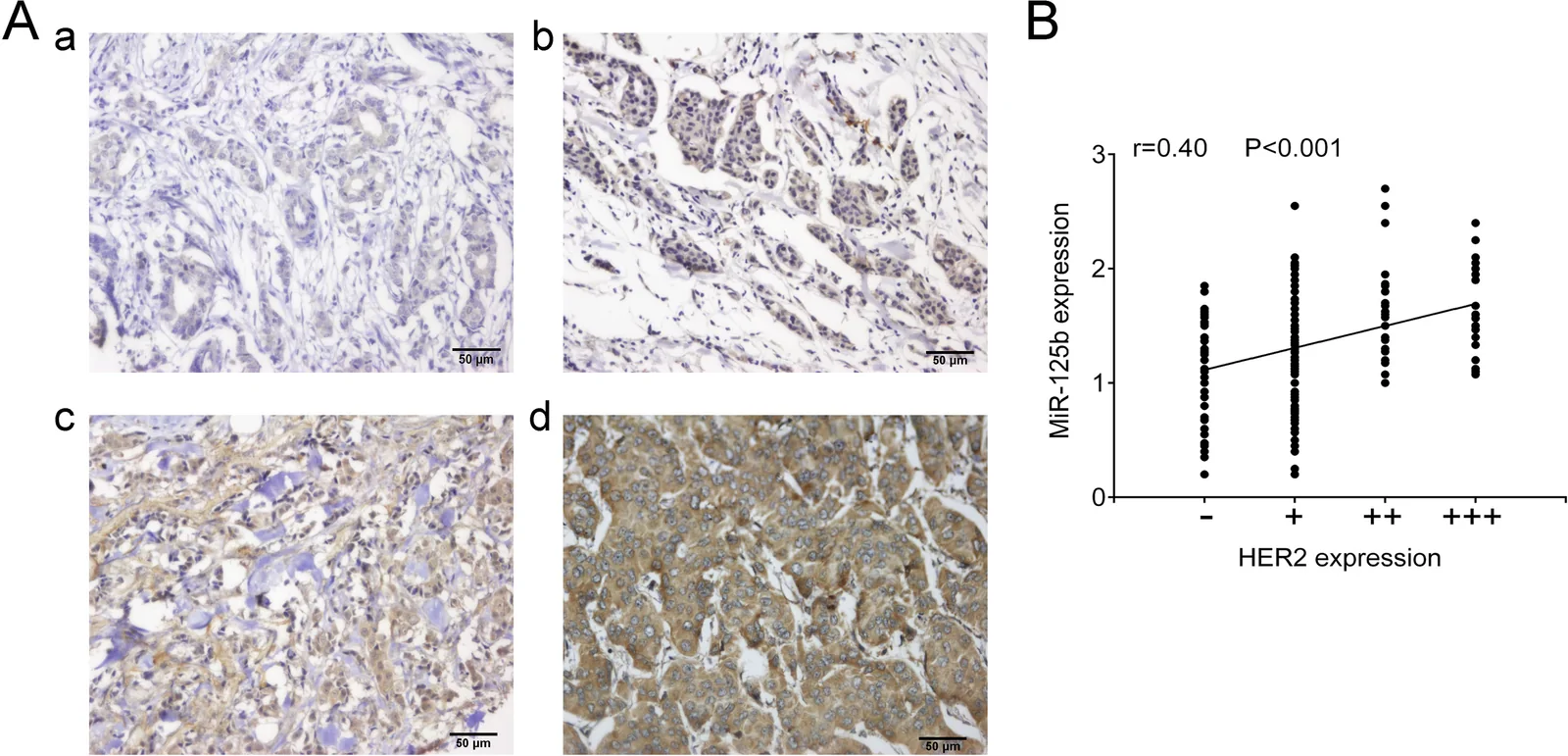
miR-125b was positively correlated with HER2 expression in breast cancer. (A) Representative images of miR-125b expression in (a) HER2 negative (-), (b) weak (+), (c) moderate (++) and (d) strong (+++) breast cancer tissues. Scale bar, 50 *µ*m. (B) Spearman's correlation analysis was used to analyze the correlation between miR-125b and HER2 in breast cancer. r=0.40, P<0.001. HER2, human epidermal growth factor 2; miR, microRNA..

